# Development of the Perinatal Depression Inventory (PDI)-14 using item response theory: a comparison of the BDI-II, EPDS, PDI, and PHQ-9

**DOI:** 10.1007/s00737-015-0553-9

**Published:** 2015-08-14

**Authors:** Benjamin B. Brodey, Sherryl H. Goodman, Ruth E. Baldasaro, Amy Brooks-DeWeese, Melanie Elliott Wilson, Inger S. B. Brodey, Nora M. Doyle

**Affiliations:** TeleSage, Inc., 201 E. Rosemary St., Chapel Hill, NC 27510 USA; Emory University, PAIS Building, 36 Eagle Row, Atlanta, GA 30322 USA; SAS Institute Inc., 100 SAS Campus Drive, Cary, NC 27513-2414 USA; Mental Health Partners, 1333 Iris Ave., Boulder, CO 80304-2226 USA; NetSmart Technologies, Inc., 4950 College Boulevard, Overland Park, KS 66211 USA; University of North Carolina at Chapel Hill, Greenlaw 211, Chapel Hill, NC 27599 USA; Department of Obstetrics and Gynecology, University of Oklahoma-Tulsa, 4444 E. 41st Street, Tulsa, OK 74135 USA

**Keywords:** Depression, Perinatal, Postpartum, IRT, PDI-14

## Abstract

The objective of this study is to develop a simple, brief, self-report perinatal depression inventory that accurately measures *severity* in a number of populations. Our team developed 159 Likert-scale perinatal depression items using simple sentences with a fifth-grade reading level. Based on iterative cognitive interviewing (CI), an expert panel improved and winnowed the item pool based on pre-determined criteria. The resulting 67 items were administered to a sample of 628 pregnant and 251 postpartum women with different levels of depression at private and public sector obstetrics clinics, together with the Beck Depression Inventory (BDI-II), Edinburg Postpartum Depression Scale (EPDS), and the Patient Health Questionnaire (PHQ-9), as well as Module A of the Structured Clinical Interview for DSM-IV Diagnoses (SCID). Responses were evaluated using Item Response Theory (IRT). The Perinatal Depression Inventory (PDI)-14 items are highly informative regarding depression severity and function similarly and informatively across pregnant/postpartum, white/non-white, and private-clinic/public-clinic populations. PDI-14 scores correlate well with the PHQ-9, EPDS, and BDI-II, but the PDI-14 provides a more precise measure of severity using far fewer words. The PDI-14 is a brief depression assessment that excels at accurately measuring depression severity across a wide range of severity and perinatal populations.

**Electronic supplementary material:**

The online version of this article (doi:10.1007/s00737-015-0553-9) contains supplementary material, which is available to authorized users.

## Introduction

Between 320,000 and 1,000,000 women in the USA experience symptoms of perinatal depression each year (Bennett et al. 2004; Gavin et al. 2004). Davis (2013) reported a period prevalence for postpartum depression of 10 %, but according to Lobato (2011), it can reach as high as 24.3 % in some underprivileged populations. Moreover, even in privileged circumstances, universal screening rarely occurs and more than half of women with perinatal depression go undetected (Delatte et al. 2009; Boyd et al. 2005; US Department of Health and Human Services 2000). As defined in both the fourth and fifth editions of the Diagnostic and Statistical Manual of Mental Disorders (i.e., DSM-IV and DSM 5), a diagnosis of Major Depressive Disorder (MDD) can manifest itself heterogeneously and can represent a wide range of severity (APA, 1994, 2013). One postpartum woman might experience *sadness*, *guilt, weight loss*, *tiredness*, and *insomnia,* symptoms that are all common in the postpartum period and which might represent very mild symptoms of depression. A second postpartum woman might experience *worthlessness*, *hopelessness*, *anhedonia*, *indecisiveness*, and *recurrent thoughts of death*, a cluster that should concern any clinician. Despite being very different, both of these women have the requisite five symptoms necessary to meet criteria “A” for the same MDD diagnosis. Given its prevalence, the importance of providing timely mental health treatment (Stein 2014), the potential adverse consequences of unnecessary pharmacologic interventions, and the heterogeneity represented by a diagnosis of MDD, there is great need for a reliable perinatal instrument, one that assesses not just the presence or absence of the MDD diagnosis, but one that accurately measures the *severity* of perinatal depression, particularly as it relates to impairment (Goodman 2010).

Although several scales are available, some of which are commonly used in both clinical and research settings, there is no current consensus on the most useful or psychometrically sound instrument. Each of the commonly used scales has notable strengths and weaknesses.

First, most existing instruments were not developed incorporating the benefitted of Modern Measurement Theory (MMT) methods including Item Response Theory (IRT) which does not assume that each item is equally related to depression. Instead, by allowing items to differ in their severity and “reliability,” the use of MMT can result in much more precise measurements of depression severity. Though Logsdon (2009) retrospectively subjected the Edinburg Postpartum Depression Scale, or EPDS (Cox et al. 1987), to IRT analysis, none of the most widely used instruments were developed using IRT. Most assessments were developed using Classical Test Theory (CTT) without the benefits of MMT and generally without the benefits of Cognitive Interviewing (CI). MMT allows for the better identification of highly “informative” items, which can increase accuracy in differentiating levels of depression (Guedeney et al. 2000). CI facilitates the development of easy-to-understand items that are interpreted quickly and unambiguously. Used together, CI and MMT are not just validation strategies: they represent more fundamental techniques for determining which items to include in an assessment. MMT-based instruments can be designed to minimize “differential item functioning” (DIF) by eliminating items that function differently—that is, yield different responses in different populations (e.g., pregnant vs. postpartum, patients seen at public vs. patients seen at private clinics, white vs. non-white). Like traditional CTT instruments, MMT-based instruments can be used for assessing simple dichotomous variables such as the presence or absence of MDD; however, they are ideal for assessing severity, a continuous variable.

Second, most measures used to assess depression in the perinatal period were designed to assess general depression for both men and women not perinatal depression, although several of them have subsequently been validated for use in perinatal populations, this is particularly true of the Beck Depression Inventory–II (BDI-II) and the Patient Health Questionnaire-9, or PHQ-9 (Beck et al. 1961; Beck et al. 1996; Kroenke et al. 2001). As a result, these instruments include experiences that may be common both before and after pregnancy (e.g., changes in appetite and changes in sleep) but are experienced independent of depression. This casts doubt on the content validity of these scales in perinatal populations. Other instruments, particularly the Edinburgh Postpartum Depression Scale (EPDS) and the Postpartum Depression Screening Scale (PDSS), were initially developed for use during the *post*partum period and not for pregnancy (Cox et al. 1987; Beck and Gable 2000) or designed for pregnancy without consideration of the postpartum period (e.g., the Antenatal Screening Questionnaire (ASQ), (Appleby et al. 1994)). Again, the lack of focus on these populations during development raises questions about the appropriateness of these measures for use during pregnancy or, depending on the scale, postpartum.

The literature currently lacks a depression scale specifically designed and developed with input from and for use with both pregnant and postpartum women. The need for such an instrument has recently been highlighted by findings suggesting that “postpartum” depression frequently has its onset during pregnancy (Guedeney et al. 2000).

Third, several instruments, such as the BDI-II, the EPDS, and the ASQ, use a variety of long response options. For example, question 8 on the BDI-II, option “0” is “I don’t criticize or blame myself more than usual” (Cox et al. 1987; Beck et al. 1996; Appleby et al. 1994). On the EPDS, question 6, response option 1 states that “No, most of the time I have coped quite well.” Long response options like these that change with each question increase the reading and interpretive efforts needed to complete a survey relative to a fixed, repeated Likert-scale with one-word response options. Fourth, some of the scales contain idiomatic language and individual words that confuse people in different English-speaking countries and which make translation into other languages imprecise. For example, an EPDS item uses the phrase “things have been getting on top of me.” This British idiomatic phrase is intended to mean “overwhelmed,” but it does not transfer well to American settings and a literal interpretation loses this meaning. One of the BDI-II (Beck et al. 1996) items uses the word “restless” which can mean either “agitated,” “anxious,” or “unable to focus”; to others, it may mean (more literally) “without rest” or “sleepless.”

Fifth, the BDI-II and the EPDS items and responses are fairly long, with 812 and 264 words, respectively (Cox et al. 1987; Beck 1996). This creates difficulties for patients with low literacy and may affect administration time.

Finally, none of the existing instruments were developed and calibrated to measure depression similarly whether in antenatal/postpartum, private/public care settings, or white/non-white populations.

We developed and evaluated our new perinatal depression inventory to address *all* of these concerns. The primary aim of this work was to create a brief, highly informative instrument that could be used continuously throughout the antenatal and postpartum periods, such that when a perinatal woman reads an item in the new Perinatal Depression Inventory, she will immediately know the response that is correct for her without needing to interpret the intent of the question.

## Materials and methods

### Item development

There were two stages to item *development*. First, a careful review of 25 published measures of either general, postpartum, or antenatal depression was undertaken as well as a review of DSM-IV-TR (American Psychiatric Association 1994) depression criteria and text. From these measures, items representing all core concepts as well as *DSM* sub-criteria for MDD were identified and grouped by the isolated symptom or concepts that they appeared to represent—e.g., hopelessness, worthlessness, concentration, or indecision). Since the individual criteria in DSM 5 (APA 2013) are identical to those of DSM-IV-TR with the exception that “hopelessness” was added, we have adhered to the numbering system used in the DSM 5.

In the second stage of item development, we composed our own items representing each of these concepts/symptoms to address the concerns noted previously. To ensure the consistency and clarity of the new items, we developed the following guidelines. First, items were constructed to fit a “past 7 days” time frame, which, relative to longer retrospective time frames, minimizes reporting discrepancies (Appleby et al. 1994). Second, items had to fit a single five-point Likert-scale (“Never, Rarely, Sometimes, Often, Always”). The advantage of this approach is that the reader becomes familiar with the response set and does not need to relearn the responses with each new question. Third, to the greatest extent possible, items could not include multiple concepts (e.g., item 6 on the PHQ-9 which describes “Feeling bad about yourself—or that you are a failure or have let yourself or your family down”), idiomatic/culture-specific language (e.g., “jumping out of my skin” from the PDSS), or words that have differing abstract and concrete interpretations (e.g., “downhearted” *or* “blue”). Fourth, items were developed to reflect a fifth-grade reading level (e.g., we avoided words such as “discouraged,” “particularly,” “worthwhile,” “experienced,” and “fatigued” that can be found in the BDI-II (Fava et al. 2009)). Fifth, we avoided using negatives where misunderstanding the negative might reverse the meaning of the question (e.g., “I feel no more tired or fatigued than usual” from the BDI-II). This process resulted in a pool of 159 items.

In the first stage of item *reduction*, the 159 items were independently reviewed and rated by a panel of experts, including a psychiatrist, three PhD-level clinical psychologists, a registered nurse, a certified nurse midwife, and two women who had suffered from perinatal depression. Our goal was to retain a wide range of concepts while eliminating similar but inferior items. Items were rated on a 1–3 scale for clarity and a 1–3 scale for centrality to the domain of perinatal depression (higher is better). Items were retained if they were deemed to be clear and central as evidenced by an average score of at least 2.6 on both scales. This cutoff both enabled us to assure that the items were strong, while it allowed us to retain a number of items for which CI was feasible. For very similar items, the lower scoring item was removed from the item pool. This reduced the item pool to 86 items, a number that made CIs feasible.

The second stage of item reduction consisted of CIs, administering the 86 items to 20 pregnant and 10 postpartum women who had scores on the EPDS in one of the three categories: greater than 12 (a validated score indicating clinically significant levels of depression) (5 women), 9–12 (indicates possible depression) (7 women), and less than 9 (low depression symptom levels) (18 women). Each CI was conducted by a master-level mental health clinician and took approximately 90 min to complete. Interviews were audio-recorded for later coding. All participants responded to the entire set of items. In all instances, the interviewers provided ample opportunity for open exploration of items and responses. CIs identified 19 items that were misinterpreted in a way that led to an inaccurate response by 20 % or more of participants. These were omitted from further consideration, leaving 67 items that were included in the quantitative validation described in the next section.

### Participants and procedures

In order to validate the item pool, reduce its size, and assure uniform functioning of items across several demographic populations, we studied a sample of 879 pregnant or postpartum women. The 628 pregnant women were on average 27.06 years old (SD = 5.91). The 251 women who had given birth within the previous 150 days were on average 28.86 years old (SD = 5.90). Both groups were recruited from a private obstetrics clinic in Atlanta, GA, and a public obstetrics clinic in Tulsa, OK. The sample of pregnant women represented a wide range of racial/ethnic identities including Hispanic ethnicity (7.1 %); African-American (36 %); White (42 %); Asian, Native American, Native Hawaiian, Pacific Islander, or multi-racial (18 %); and unknown (4 %). The sample of postpartum women was similarly diverse: Hispanic ethnicity (8.6 %); African-American (39 %); White (44 %); Asian, Native American, Native Hawaiian, Pacific Islander, or multi-racial (12 %); and unknown (5 %).

Participants were patients at the respective clinics but came into the obstetrics clinic for a single research visit. After completing the informed consent process, participants were given self-report items and instruments in paper format: the 67 newly developed perinatal depression items along with the EPDS, PHQ-9, and BDI-II (Cox 1987; Beck 1996; Kroenke 2001). An experienced interviewer administered the mood module (Module A) of the Structured Clinical Interview for DSM-IV-TR (SCID) to identify the presence of a current MDD (APA 1994; First 1995). These scales were chosen because (a) the EPDS is the most frequently used perinatal depression scale (Boyd 2005; Cox 1987); (b) the BDI-II is also commonly used to screen for postpartum depression (Beck 1996; Seehusen 2005); and (c) the PHQ-9 is both commonly used and has the items most closely paralleling the DSM-IV diagnostic criteria for MDD (Kroenke 2001; Spitzer 1999). To control for order effects, the order of the four paper survey instruments was randomized. Additionally, half of the women were randomly assigned to complete the SCID interview followed by the paper surveys, whereas the other half completed the paper-surveys followed by the SCID interview (First 1995).

### Measures

In addition to the 67 candidate items, participants completed the following:

1. The EPDS (Cox 1987) consists of ten items (264 words) with response options that vary greatly between items but which are scored from 0 to 3. The total score is the sum of all item responses, ranging from 0 to 30.
2. The PHQ-9 (Kroenke et al. 2001) contains 220 words and is based on the DSM-IV diagnostic criteria, with response categories ranging from 0 (“Not at all”) to 3 (“Nearly every day”). The total score is the sum across items and can range from 0 to 27.
3. The BDI-II (Beck 1996) consists of 21 items (812 words) with lengthy response options that vary with every question. The total score is a sum of the item scores, with a potential range from 0 to 63.
4. Module A of the SCID (First 1995) for DSM-IV-TR is a gold standard for the diagnoses of depressive disorders, Module A of the SCID was administered by an experienced MA-level clinician to identify the presence/absence of a current major depressive episode (MDE).

### Statistical analyses

Multiple group analyses were planned for three demographic variables: (A) pregnancy status (pregnant/postpartum); (B) clinic type (public/private clinic); and (C) race (white/non-white)—race was dichotomized as white/non-white due to the low number of non-white, non-black participants, e.g., Asians, Native Americans, and Pacific Islanders. To facilitate the planned analyses, item response frequencies were reviewed to identify items for which any group had fewer than five responses in a response category. For a few such extreme items, including those relating to suicide, the responses were collapsed in all groups so that the response category with fewer than five observations was recoded into the adjacent response category.

Next, the dimensionality of the item set was examined in order to assure that the statistical model used to score the items appropriately modeled the relationships among the items. Because the items used ordinal response categories, dimensionality was assessed using categorical confirmatory factor analysis (CCFA) using diagonally weighted least squares estimation, as implemented in Mplus 6.11 (Kroenke et al. 2001). To assess the fit of each model, we examined the comparative fit index (CFI >0.95 indicates good fit), the Tucker-Lewis fit index (TLI >0.95 indicates good fit), and the root mean squared error of approximation (RMSEA <0.08 indicates adequate fit).

IRT parameter estimates were used to identify items that were a poor representation of depression (e.g., low information and/or discrimination). Differential item functioning (DIF) analyses identified items that performed differently across any of the three demographic variables. DIF was assessed using Wald tests. DIF analyses calculate test statistics for each item and each set of parameters for that item (i.e., slopes, intercepts) to determine whether item parameters differ between any of the demographic groups. The Benjamini-Hochberg (Muthén and Muthén 2011) false discovery rate procedure was applied to the resulting Wald test values to control type I error. If the statistical test is significant, DIF is suspected to exist among the tested groups. The IRT and DIF analyses were conducted in flexMIRT version 1.88 (Cai 2012).

The test information function (TIF) and expected standard error curve (SEC) were plotted to demonstrate the precision of the final Perinatal Depression Inventory (PDI) scores. To assess the convergent validity of the final PDI items, we calculated correlations between it and the three other depression scales. In addition, receiver operating curve (ROC) analyses were used to determine optimal cut-off scores on the new scale, to distinguish between individuals with and without MDE diagnoses as measured by the gold standard SCID interview. The correlation and ROC analyses were conducted in SAS 9.2 (SAS Institute Inc 2008).

IRT analyses were also performed on the EPDS, BDI-II, and PHQ-9 using models from the existing MMT literature for these instruments.

## Results

### Dimensionality and IRT modeling

We initially performed a unidimensional IRT analysis on the 67 items in order to further reduce the item pool. From the 67 items, we selected the two most informative items representing each of ten DSM-5 symptom criteria for MDD. From the remaining 20 items, we selected the overall most informative items, maximizing the total area under the item information curve (IIC) in order to select a total of 40 items for more detailed evaluation. Forty seemed to be a small enough sample that the items would be amenable to IRT analysis yet large enough that they would still have sufficient coverage of the necessary topics. For these 40 items, both a unidimensional CCFA model and a bifactor model were fit to the PDI candidate items to assess dimensionality. With the bifactor model, all items loaded onto a general “depression” factor as well as onto no more than one additional specific factor. These specific factors correspond to the specific symptom, DSM sub-criterion, or distress/impairment criterion for MDE in the DSM-IV-TR. For sub-criteria with only two items, the specific factor loadings were constrained to equality for identification of the factor or a residual correlation was specified between the two items within the sub-criterion. The unidimensional model was found to have acceptable model fit (*χ*^2^ = 6249.92, *df* = 740, *p* < 0.001; CFI = 0.94; TLI = 0.94; RMSEA = 0.09), while the bifactor model was found to have good model fit (*χ*^2^ = 2838.17, *df* = 707, *p* < 0.001; CFI = 0.98; TLI = 0.97; RMSEA = 0.06). Using the DIFFTEST procedure available in Mplus (Kroenke et al. 2001), the unidimensional model was associated with a statistically significant decrease in model fit relative to the bifactor model, *X*^2^_diff_ (33) = 2298.8, *p* < 0.001. That is, the bifactor model statistically better represented the data relative to the single factor model and, therefore, the bifactor model was used in subsequent analyses.

### Differential item functioning

We examined DIF to determine whether items needed to be eliminated due to not performing equivalently across the three pairs of demographic variables: (A) pregnant/postpartum, (B) public/private clinic, and in (C) white/non-white. DIF is generally undesirable. The most commonly observed type of DIF in this study, *threshold*, affected the difficulty parameter with respect to the type of clinic at which the participant was receiving obstetric care (i.e., public versus private). Generally, threshold DIF means that members of one group tended to score higher or lower on an item relative to members of the other group. *Slope* DIF means that an item was interpreted in a qualitatively different way by members of one group relative to the other, i.e., item responses were either more or less informative (reliable) across groups. Although only six items were found to be invariant across all three pairs, none of the items showed slope DIF and the remaining items did not display meaningful threshold DIF (i.e., the weighted root-mean-square error (WRMSE)) was less than 0.08.

Based on the results from the IRT and DIF analyses on the 40 candidate items, further items were eliminated. First, all items with meaningful DIF were excluded. Second, all comparatively uninformative items, items with low slopes below 1.0, were excluded. Third, the items were divided into groups representing the DSM-IV-TR (APA 1994) sub-criteria and their internal concepts. The single most informative item in each sub-criterion was included. In the instances where there were two highly informative items within a sub-criterion, the second most informative item was also selected for inclusion in the scale. For criterion 1.A., we included a third item (“I felt irritable”), since irritability as a symptom of MDD may not be limited to children; some adults may experience irritability rather than a feeling of sadness (Fava 2009; Williamson 2014). Lastly, we selected three items representing criterion B: “clinically significant distress or impairment.” We chose to do this because endorsement of criterion B is required to identify a MDE and also an important indicator of a need to intervene clinically. These three items were all highly informative, according to our criteria, and all three had slopes above 2.12. Because we chose to eliminate all items with a slope below 1.0, items representing three sub-criteria were excluded from the PDI-14 (1) “insomnia or hypersomnia,” (2) “significant weight loss or weight gain,” and (3) “psychomotor agitation or retardation.” While it would have been desirable to represent these sub-criteria, given their centrality in the DSM, they were essentially uninformative statistically. The resulting PDI has 14 items (137 words) (Table 1 ESM).

We also looked at DIF on the EPDS, BDI-II, and PHQ-9. The BDI-II showed the most DIF, with over 40 % of the items exhibiting significant DIF in at least one of the tested grouping variables. The DIF in the PHQ-9 was notable in that slope DIF was as prevalent as threshold DIF.

### Instrument scoring

Because the Perinatal Depression Inventory (PDI)-14 was developed using IRT, computer-assisted automated IRT scoring is optimal as IRT scores are more accurate and precise than sum scores. IRT scoring provides the greatest differentiation among individuals and within individuals over time, relative to a simple sum score (Appleby 1994). Computer-generated scores are easily obtained when administering the PDI-14 electronically or by entering the responses into an electronic scoring algorithm retrospectively, but are unavailable for real-time scoring of a paper version of the PDI-14.

In order to provide optimal scores when computer-scoring is not feasible, we have ensured that the PDI-14 can be used with a simple sum score just like the EPDS and BDI-II and PHQ-9. In addition, these sum scores can also be compared to IRT scores and corresponding standard scores on the other three instruments. We created a table to convert sum scores into IRT-based, probability-weighted scores. This table equates sum scores to the standard IRT *z*-metric using a probability-weighted scoring algorithm that takes into account the probability in our clinical sample of each response pattern that can result in a particular sum score and then weights the probability of each response pattern to derive the most likely theta-based (IRT *z*-metric) score (Cai 2012).

Converting summed scores to IRT-metric scores using conversion tables permits researchers and clinicians to quickly obtain scores on a common metric with known reliabilities at the time of administration and without software (see Table 2). The conversion table may also increase the precision of scores because they are based on the observed correspondence of sum scores and IRT scores in our sample.

### Instrument validity

Since some of the items selected for the PDI-14 were found to exhibit statistically significant DIF, additional analyses were conducted to determine if the DIF was practically meaningful. To examine the practical significance of the detected DIF in the PDI-14, normal-weighted RMSE (WRMSE) values were calculated for each PDI-14 item and for the scale as a whole by comparing the expected score functions from parameters that incorporated DIF to those that ignored all DIF and assumed item parameter invariance across the three demographic variables. As may be seen in Table 1, the item WRMSE values are all below 0.08 and the scale WRMSE is at or below 0.40 for all groups, indicating that expected scores from a model accounting for DIF and the expected scores from a model *not* accounting for DIF are quite similar. These values provide evidence that the detected DIF is of little practical significance (Table 2).

**Table 1:** Weighted root mean squared error (WRMSE) values for the PDI-14 expected item and scale score functions by group

Item	Pregnant				Postpartum			
	White		Non-White		White		Non-White	
	Public	Private	Public	Private	Public	Private	Public	Private
	*N* = 197	*N* = 65	*N* = 241	*N* = 125	*N* = 53	*N* = 57	*N* = 71	*N* = 70
1	0.009	0.049	0.009	0.049	0.009	0.049	0.009	0.049
2	0.007	0.055	0.007	0.055	0.007	0.055	0.007	0.055
3	0.007	0.017	0.007	0.017	0.007	0.017	0.007	0.017
4	0.030	0.030	0.005	0.005	0.030	0.030	0.005	0.005
5	0.010	0.010	0.019	0.019	0.010	0.010	0.019	0.019
6	0.009	0.060	0.009	0.060	0.009	0.060	0.009	0.060
7	0.012	0.012	0.012	0.012	0.012	0.012	0.012	0.012
8	0.025	0.079	0.024	0.032	0.025	0.079	0.024	0.032
9	0.006	0.033	0.006	0.033	0.006	0.033	0.006	0.033
10	0.005	0.015	0.005	0.015	0.005	0.015	0.005	0.015
11	0.002	0.002	0.002	0.002	0.002	0.002	0.002	0.002
12	0.007	0.007	0.007	0.007	0.007	0.007	0.007	0.007
13	0.014	0.014	0.014	0.014	0.014	0.014	0.014	0.014
14	0.012	0.056	0.012	0.056	0.012	0.056	0.012	0.056
PDI-14 scale score	0.068	0.400	0.025	0.332	0.068	0.400	0.025	0.332

**Table 2:** PDI-14 sum score to IRT EAP score conversion table

PDI-14 sum score	PDI-14 EAP score	Expected SE	PDI-14 sum score	PDI-14 EAP score	Expected SE
0	−1.39	0.41	22	0.55	0.25
1	−1.49	0.42	23	0.64	0.25
2	−1.68	0.46	24	0.73	0.25
3	−2.07	0.64	25	0.81	0.25
4	−1.70	0.53	26	0.90	0.26
5	−1.46	0.48	27	0.99	0.26
6	−1.24	0.44	28	1.09	0.26
7	−1.05	0.40	29	1.18	0.26
8	−0.89	0.38	30	1.28	0.27
9	−0.74	0.35	31	1.38	0.27
10	−0.61	0.34	32	1.49	0.28
11	−0.49	0.32	33	1.61	0.29
12	−0.38	0.31	34	1.74	0.30
13	−0.27	0.30	35	1.88	0.31
14	−0.17	0.29	36	2.04	0.33
15	−0.07	0.28	37	2.23	0.37
16	0.02	0.27	38	2.46	0.42
17	0.12	0.26	39	2.78	0.52
18	0.21	0.26	40	2.55	0.38
19	0.29	0.26	41	2.46	0.34
20	0.38	0.25	42	2.38	0.33
21	0.47	0.25			

Very few participants in our sample had a BDI-14 sum score above 42, we were, therefore, unable to compute correspondences beyond 42

**Fig. 1:**
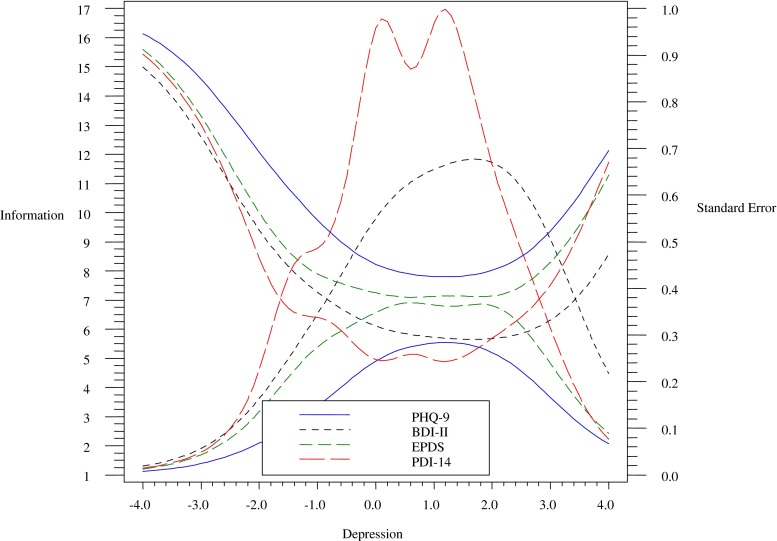
The test information function and standard error curves of the PDI-14, the BDI-II, the PHQ, and the EPDS

To provide evidence for the concurrent validity of the PDI-14, correlations with the three other depression measures were calculated. The PDI-14 was found to correlate strongly with the BDI-II (*r* = 0.82, *p* < 0.001), EPDS (*r* = 0.81, *p* < 0.001), and PHQ-9 (*r* = 0.77, *p* < 0.001). In addition, to demonstrate that the PDI-14 provides excellent information across a wide range of depression severity, Fig. 1 shows the test information function (TIF) and standard (SE) error function of the scale which illustrates the accuracy of the assessment by detailing its precision across theta values, differing levels of depression severity, while allowing for varying degrees of standard error (as opposed to assuming equal error across interviewees), respectively. As can be seen in Fig. 1, the PDI-14 has high test information and good precision from SD −1.0 to 2.5. That is, the PDI-14 accurately differentiates severity of depression across a very broad range of perinatal depression severities.

Finally, for all of the instruments, ROC analyses were used to calculate optimal cut-off criteria for the identification of individuals likely to meet DSM-5 criteria for a current MDE. The PDI-14 was found to have an area under the curve of 0.90, demonstrating that it is both sensitive and specific. The results of the ROC analyses indicate that 0.21 in the IRT metric is the optimal cut-off to indicate likelihood of a MDE for the PDI-14. This cut-off produced a sensitivity of 0.83, with a specificity of 0.78 for MDE. These results provide evidence for the ability of the PDI-14 to discriminate between healthy individuals and those experiencing a major depressive episode. Optimal cutoffs for the comparison instruments were also determined. A BDI cutoff of 13 yielded a sensitivity of 0.82 with a specificity of 0.75. A cutoff of 11 on the EPDS yielded a sensitivity of 0.82 and a specificity of 0.81. A cutoff of 0.7 on the PHQ-9 yielded a sensitivity of 81 and a specificity of 0.79.

## Discussion and conclusions

We set out to develop a brief self-report perinatal depression inventory that would be easy to understand and that would accurately measure the *severity of depression* in a number of populations. We succeeded in our goals by creating an assessment that appears to measure the severity of perinatal depression more precisely than the BDI-II, the EPDS, and the PHQ-9. Based on their relative word counts including the question and response options for each item, the PDI-14 (with 137 words) is only 17 % of the length of the BDI-II, just over half (52 %) of the length of the EPDS, and 62 % as long as the PHQ-9. The PDI-14 was constructed to minimize DIF among antenatal/postpartum women, as well as between white/non-white, and public/private clinic patients. This is important because it means that using the PDI-14, severity can be measured similarly across these populations without the need to make mathematical adjustments to scores. The practical implication of this is that the PDI can be administered to all perinatal women in a waiting room and can be scored with confidence that the score represents an accurate assessment of depression severity for that individual. Based on the rigorous methodology used to develop the assessment, we believe it offers a strong alternative to the commonly used measures of perinatal depression.

The results of this study also provide some interesting insights: although we cannot say that depression during pregnancy and in the postpartum period represent the same phenomenon, we were able to select a group of items to which women who are equally depressed respond similarly whether they are antenatal or postpartum, and, further, whether they seek care in the private or public sector and whether they are white or non-white. This provides an opportunity to look more objectively at the severity of depression independent of these potential confounders (e.g., the PDI-14) and enables us to say that a given antenatal woman and a given postpartum woman are *equally* depressed even though their circumstances may vary greatly. Similarly, it enables us to follow an individual woman from the antenatal to the postpartum period and to track the severity of depression through this entire period, confident that the measurement is equivalent. This is particularly important since we know that postpartum depression often has its onset in pregnancy or even prior to pregnancy (O’ Hara and Wisner 2014). Use of the PDI-14 may facilitate research into the longitudinal features of perinatal depression.

We chose to use the widely adopted “past 7 days” time frame for the PDI-14 to increase reliability relative to longer reporting periods and to assure that the assessment would be useful in outcomes tracking, such as in clinical trials. The EPDS uses the same “past 7 days” time frame, while the BDI-II and the PHQ-9 both use a “2-week” time frame. In the DSM description of MDD, it states that “symptoms have been present during the same 2-week period.” This might suggest that asking about a 2-week time frame would yield greater specificity for MDD; however, the ROC for the PDI-14 is essentially equivalent to the ROCs for the PDI-14, BDI-II, EPDS, and PHQ-9. This suggests that in assessing antepartum and postpartum MDD, asking about a 2-week time period rather than a 1-week time period is not necessary to achieve high sensitivity and specificity for MDD. This finding may have implications for the future definition of antenatal and postpartum depression.

The results of the concurrent validity studies of the PDI-14 against the EPDS, PHQ-9, BDI-II, and SCID suggest that the PDI-14 is measuring a construct that is similar but not identical to the constructs measured by previously developed depression measures. Representing all of the DSM-5 sub-criteria and concepts of depression in the scale might have been desirable but might also have increased the likelihood of false positives in the perinatal population. Even though items associated with changes in sleep, changes in appetite or weight, and psychomotor agitation or retardation fit the bifactor analytic model, they were not informative, as indicated by the IIC which all had slopes of less than one, and were therefore dropped from the final scale. In other words, these common symptoms often associated with depression also appear to be common in non-depressed perinatal women and therefore did not differentiate the severity of depression. Items on energy and irritability were included in the inventory, but were only slightly informative, having lower slope values than other retained items. These findings might also suggest that perinatal depression would be more clearly defined if it had unique diagnostic criteria rather than sharing all criteria with MDD (Class 2013).

Given the excellent TIF of the PDI-14 relative to the other instruments, one might have expected that the ROC for the PDI-14 to be superior to the other instruments. (The item information curve (IIC) represents the ability of an item to correctly differentiate levels of depression. In general, the TIC shows the ability of the test to differentiate severity; it is the sum of the individual IICs). This, however, was not the case. The ROCs for the PDI-14, EPDS, BDI-II, and PHQ-9 were all fairly similar. We believe that this is because all of these instruments, including the PDI-14, are based upon the criteria as defined by the DSM; their ROCs are therefore limited by the heterogeneous nature of the phenomena called “MDD” as defined in the DSM. As described above, we do not consider this to represent a weakness in the PDI-14, since in the perinatal population, MDD also represents a very broad spectrum of severity. Thus, it is unlikely that even a very accurate depression measure would improve upon existing sensitivities and specificities for MDD as currently defined in the DSM. The primary goal of the PDI is to more accurately and precisely assess *the severity of perinatal depression* in order to inform treatment decisions.

### Limitations of current study

Although this study was actually conducted prior to the development of DSM-5, the criteria for major depressive episode (MDE) in DSM-5 have changed very little. As they pertain to this study, the primary change in DSM is that antenatal depression is now recognized as a component of “peripartum” depression, and “hopelessness” has now been included in sub-criterion A.1 for MDD. We independently found that “I felt hopeless.” is the *most* informative item that we tested.

### Future directions

Although test-retest reliability was not specifically tested in this study, we can infer from the high slopes exhibited by the items in the PDI-14 that they are highly discriminating. Based on these results, we can say that provided that the underlying trait of depression is stable within a given time period, the test-retest reliability should also be high during that period. Nonetheless, test-retest reliability should be measured formally in a future study.

Although it is beyond the scope of the current project, using IRT and the data from this project, it should be possible to determine quantitatively the extent to which the PDI-14, BDI-II, EPDS, and PHQ-9 do measure the same construct. To the extent that the differences are small, it will then be possible to create a conversion table for all four instruments. This table would facilitate future meta-analyses.

## Electronic supplementary material

ESM 1 (DOCX 16 kb)

ESM 2 (DOCX 20 kb)
