# Supplementary material for: Development of the Perinatal Depression Inventory (PDI)-14 using item response theory: a comparison of the BDI-II, EPDS, PDI, and PHQ-9
Source: Arch Womens Ment Health. 2015 Aug 14;19:307–16. doi: 10.1007/s00737-015-0553-9 (PMC4799794; doi:10.1007/s00737-015-0553-9)
Supplement: Supplementary file 1 — (DOCX 16 kb) [file 737_2015_553_MOESM1_ESM.docx]

Appendix A. PDI-14 items and IRT item parameters.

|  |  | Difficulty | | | |
| --- | --- | --- | --- | --- | --- |
| Item | General Slope* | b1 | b2 | b3 | b4 |
| 1. I felt sad. | 3.44 | -0.89 | -0.08 | 0.89 |  |
| 2. I felt depressed. | 2.01 | -0.35 | 0.28 | 0.96 | 1.76 |
| 3. I felt irritable. | 1.12 | -1.19 | -0.38 | 0.63 |  |
| 4. I felt unhappy. | 2.29 | -0.7 | 0.15 | 1.11 |  |
| 5. I enjoyed life. | 1.51 | -0.26 | 0.6 | 1.56 |  |
| 6. I felt worthless. | 2.21 | 0.32 | 0.84 | 1.54 |  |
| 7. I felt disappointed in myself. | 2.26 | -0.39 | 0.27 | 1.17 | 1.9 |
| 8. I felt hopeless. | 4.09 | 0.08 | 0.67 |  |  |
| 9. I had difficulty keeping my mind on what I was doing. | 1.68 | -0.76 | 0.07 | 1.09 |  |
| 10. I had difficulty making decisions. | 1.16 | -0.63 | 0.2 | 1.14 |  |
| 11. I had thoughts of ending my life. | 1.35 | 1.6 |  |  |  |
| 12. I felt tired, even after resting. | 1.12 | -1.29 | -0.57 | 0.44 | 1.39 |
| 13. I felt like a failure. | 2.26 | 0.09 | 0.65 | 1.4 |  |
| 14. I was able to manage my problems. | -2.13 | -1.32 | -0.4 | 0.66 |  |

Note: item parameters are in approximate normal metric.
