# Supplementary material for: Development of the Perinatal Depression Inventory (PDI)-14 using item response theory: a comparison of the BDI-II, EPDS, PDI, and PHQ-9
Source: Arch Womens Ment Health. 2015 Aug 14;19:307–16. doi: 10.1007/s00737-015-0553-9 (PMC4799794; doi:10.1007/s00737-015-0553-9)
Supplement: Supplementary file 2 — (DOCX 20 kb) [file 737_2015_553_MOESM2_ESM.docx]

**Perinatal Depression Inventory (PDI-14)**

The questions that follow are about your general mood. In this survey, all the questions refer to how you have been feeling and what you have been doing during the past 7 days. There are no right or wrong answers to these questions.

| **In the past 7 days …** | | **Never** | **Rarely** | **Sometimes** | **Often** | **Always** |  | **TOTAL** |
| --- | --- | --- | --- | --- | --- | --- | --- | --- |
| **1.** | I felt sad. | 1 | 2 | 3 | 4 | 5 |  |  |
| **2.** | I felt depressed. | 1 | 2 | 3 | 4 | 5 |  |  |
| **3.** | I felt unhappy. | 1 | 2 | 3 | 4 | 5 |  |  |
| **4.** | I felt irritable. | 1 | 2 | 3 | 4 | 5 |  |  |
| **5.** | I enjoyed life. | 1 | 2 | 3 | 4 | 5 |  | * |
| **6.** | I felt worthless. | 1 | 2 | 3 | 4 | 5 |  |  |
| **7.** | I felt disappointed in myself. | 1 | 2 | 3 | 4 | 5 |  |  |
| **8.** | I felt like a failure. | 1 | 2 | 3 | 4 | 5 |  |  |
| **9.** | I felt hopeless. | 1 | 2 | 3 | 4 | 5 |  |  |
| **10.** | I had thoughts of ending my life. | 1 | 2 | 3 | 4 | 5 |  |  |
| **11.** | I felt tired, even after resting. | 1 | 2 | 3 | 4 | 5 |  |  |
| **12.** | I was able to manage my problems. | 1 | 2 | 3 | 4 | 5 |  | * |
| **13.** | I had difficulty keeping my mind on what I was doing. | 1 | 2 | 3 | 4 | 5 |  |  |
| **14.** | I had difficulty making decisions. | 1 | 2 | 3 | 4 | 5 |  |  |
|  |  |  |  | | ****Total** | |  |  |

**Scoring the PDI-14**

*** Items “5” and “12” are reverse scored**

****A sum score of 32 is consistent with MDE; Sensitivity 0.83, Specificity 0.78**
